# Strong Associations Exist among Oxidative Stress and Antioxidant Biomarkers in the Circulating, Cellular and Urinary Anatomical Compartments in Guatemalan Children from the Western Highlands

**DOI:** 10.1371/journal.pone.0146921

**Published:** 2016-01-20

**Authors:** María J. Soto-Méndez, Concepción M. Aguilera, María D. Mesa, Laura Campaña-Martín, Victoria Martín-Laguna, Noel W. Solomons, Klaus Schümann, Ángel Gil

**Affiliations:** 1 Center for the Studies of Sensory Impairment, Aging, and Metabolism–CeSSIAM–Guatemala City, Guatemala; 2 Department of Biochemistry and Molecular Biology II, Institute of Nutrition and Food Technology “José Mataix”, Center for Biomedical Research, University of Granada, Granada, Spain; 3 Networking Biomedical Research for Obesity and Nutrition–CIBERobn-, Madrid, Spain; 4 Thematic Networks of Cooperative Research–RETIC–, Carlos III Health Institute–ISCIII–, General Sub-Directorate for Research Assessment and Promotion and the European Regional Development Fund–ERDF–ref. RD12/0026, Madrid, Spain; 5 Molecular Nutrition Unit, ZIEL, Research Center for Nutrition and Food Science, Technische Universität München, Freising, Germany; Penn State College of Medicine, UNITED STATES

## Abstract

**Background:**

A series of antioxidant enzymes and non-enzymatic compounds act to protect cells from uncontrolled propagation of free radicals. It is poorly understood, though, to what extent and how their interaction is harmonized.

**Objectives:**

To explore associative interactions among a battery of urinary and blood biomarkers of oxidative stress and enzymatic and non-enzymatic markers of the antioxidant defense system in children from low income households.

**Methods:**

For this cross-sectional descriptive study, urine, red cells, and plasma were sampled in 82 preschool children attending three daycare centers in Quetzaltenango Guatemala. The urinary oxidative stress biomarkers studied were F2-isoprostanes and 8-hydroxy-deoxy-guanosine. Red cell enzyme activities measured were: catalase, superoxide dismutase, glutathione peroxidase and glutathione reductase. Circulating non-enzymatic antioxidants selected were: retinol, tocopherols, β-carotene and coenzymes Q_9_ and Q_10_.

**Results:**

In a Spearman rank-order correlation hemi-matrix, of 55 paired combinations of the 11 biomarkers, 28 (51%) were significantly correlated among each other (p≤ 0.05), with the strongest association being retinol and tocopherols (r = 0.697, p<0.001), and 4 associations (9%) showed a trend (p> 0.5 to ≤ 0.10). F2-isoprostanes showed the greatest number of cross-associations, having significant interactions with 8 of the 10 remaining biomarkers. Goodness-of-fit modeling improved or maintained the r value for 24 of the significant interactions and for one of the 5 borderline associations. Multiple regression backward stepwise analysis indicated that plasma retinol, β-carotene and coenzyme Q_10_ were independent predictors of urinary F2-isoprostanes.

**Conclusion:**

Numerous significant associations resulted among biomarkers of oxidation and responders to oxidation. Interesting findings were the apparent patterns of harmonious interactions among the elements of the oxidation-antioxidation systems in this population.

## Introduction

The oxidation of substrates in the mitochondria to generate ATP is associated with continuous free radical formation [1,2]. At the same time, free radicals can damage cellular and organelle membranes, the cell nucleus, and protein chains. Free radical action forms isoprostanes from lipids [3–6], 8-hydroxy-deoxyguanosine from DNA [6–8], and carbonyl-amino acids from peptide chains [9]. With respect to the origins of the marker of DNA damage, the formation seems to arise in the repair process for the damaged nucleoside of the nucleotide chain [10]. Action of redox enzymes is part of the normal cellular metabolism in living organisms [11,12]. Thus, oxidative mechanisms are used to destroy microbes within macrophages [13,14]. Renal excretion of such split products from lipids and nucleic acids converts them into urinary biomarkers of systemic oxidative stress that help to assess the protective reaction/response by antioxidant mechanisms [15,16].

The antioxidation system in the human organism is complex and geared to the suppression of uncontrolled oxidation while permitting essential and beneficial oxidation reactions [17]. A series of compounds, including dietary nutrients, have free radical quenching properties or work in conjunction with enzymatic antioxidation reactions or both [18]. Retinol and its derivatives, ascorbate, and tocopherols, as well as diverse carotenoids and co-enzymes Q [19], are among the antioxidant nutrients. Their circulating concentrations roughly reflect the corresponding nutritional status of an individual.

Finally, a series of enzymes protect the cell from oxidation reactions involving molecular or atomic oxygen [20]. These include superoxide dismutase (SOD), catalase (CAT), glutathione peroxidase (GPX) and glutathione reductase (GSR), which are the primary intercellular antioxidant enzymes that detoxify oxygen-containing free radicals produced during normal aerobic respiration [21–23]. The zinc and copper-dependent SOD transforms the O_2_^-.^into H_2_O_2_ and O_2_ [21], whereas CAT and GSR catalyze the reduction of H_2_O_2_ into H_2_O [22,24]. GPX works in concert with GSR in a system that neutralizes hydrogen peroxide [23].

In a research project entitled “Study on the normative state and inter- and intra-individual variation in growth, hematology, hydration, and markers of oxidation, infection and inflammation in pre-school children with a similar dietary intake”, we collected concurrent data in a series of two urinary biomarkers of oxidative stress, four enzymatic biomarkers of the antioxidant defense system (ADS) in red blood cells (rbc), and five circulating antioxidant nutrients from preschool children in a governmental system of daycare centers. The degree, to which the interactions of biomarkers reflect biological harmonization is the central query of our companion article on the interaction of immunological biomarkers in this population sample [25], which is included as supplemental material (**S1 Article**). We use parallel analytical approaches of cross-associations with linear and non-linear correlations and backward elimination multiple-regressions to quantify the magnitude of interactions. We present here the result of an exploration of the mutual interplay and interactions of biomarkers of oxidative stress and the ADS in the context of variable environmental and genetic situations within the stabilizing influence of a common institutional dietary offering.

## Materials and Methods

### Study Design

This study was a descriptive, cross-sectional field research on the variation and association among oxidative stress and antioxidant biomarkers.

### Setting and Subjects

The setting for this research project was the Western Highlands of Quetzaltenango, located 136 miles from Guatemala City at 8717 ft above the sea level [26]. Attendees at three daycare centers’ from the SOSEP (*Secretaría de Obras Sociales de la Esposa del Presidente* {Secretariat of the Beneficial Works of the First Lady}) system were assigned to participate in this study. Center A in *La Esperanza* was the semi-urban setting, located 2 miles away from downtown Quetzaltenango; Center B in *La Puerta del Llano* was located in a marginal-urban setting in the outskirts of the city of Quetzaltenango; and Center C was a rural setting situated 15 miles away from Quetzaltenango in *La Estancia*, San Martín Chile Verde. Almost all children attending the centers were of Maya indigenous ethnicity. However, some of the living habits, pastimes and physical characteristics varied between centers because of the infrastructure and the environment of each location.

### Recruitment and Enrolment

#### Inclusion Criteria

To be enrolled in the study children had to be attending one of the selected SOSEP Centers, be aged 2 to 7 years, and maintaining an attendance of at least 80% during the 8 weeks of the fieldwork. Furthermore, subjects had to be apparently healthy and with no dietary restrictions related to the foods offered by the 8-week rotating menu from the SOSEP system.

#### Exclusion Criteria

Children who did not adhere to the urine collection routine, who refused to participate in the study or whose parents did not sign the consent form were excluded.

#### Ethical Considerations

The study protocol was approved by The Human Subjects Committee of the Center for the Studies of Sensory Impairment, Aging and Metabolism (CeSSIAM) and was registered at clinicaltrials.gov as NCT02203890. A parent or guardian signed the written consent form. The SOSEP’s director for the Quetzaltenango area had authorized the study. The diet offered to the children was complemented when required, in order to provide all food items on the menu. STROBE statement for this article is included as a supporting information file (**S1 STROBE Checklist**)

### Collection, Handling and Storage of Biological Samples

We collected 24-h urine samples and a 5 mL blood sample during the last of the 8 study weeks. We started urine collection at each daycare center when a child arrived (between 7:00 and 8:00 a.m.) with SOSEP personnel assisting for the collection, using BD Vacutainer® No.364999 plastic 24-h collection container (Becton-Dickinson, New Jersey, USA). After training, parents continued the collection at home. Urine collection was finished at the center, 24 h after initiation. The collection process was repeated if there was a suspicion that it was incomplete.

The collection was taken to the laboratory, where the entire urine sample was agitated in order to obtain homogeneity before aliquoting. One aliquot was stored at -80°C for 37 to 46 weeks before being sent to Granada, Spain, on dry-ice for measurement of oxidative biomarkers. The other aliquot was stored at the same temperature in Guatemala for other measurements that were held in the country.

An experienced phlebotomist collected blood samples using BD Vacutainer® 4 mL tubes with EDTA (No.367861) and Safety-Lok^TM^ deposable needles (No.367281). Samples were centrifuged to separate red blood cells from plasma; both were stored in Nalgene® Cryogenic Vials (No.5000-0012) at -80°C until shipment to the Institute of Nutrition and Food Technology, Center of Biomedical Research, University of Granada, Granada, Spain in order to determine antioxidant enzymes activity in red blood cells and antioxidant nutrients in plasma.

### Laboratory Assays and Analyses

Plasma retinol, tocopherols, ß-carotene, and coenzymes Q_9_ and Q_10_ (Co-Q_9_ and Co-Q_10_) were determined with high-performance liquid chromatographic (HPLC) methods using a 100-microliter aliquot of plasma sample previously deproteinized with 1-propanol [27] (CV intra-day 6.1%, CV inter-day 3.8%, LOD 0.012 mg/mL for retinol; CV intra-day 2.7%, CV inter-day 5.6%, LOD 0.062 mg/mL for tocopherols; CV intra-day 6.7%, CV inter-day 13.0%, LOD 0.012 mg/mL for β-carotene; CV intra-day 1.5%, CV inter-day 4.5%, LOD 0.012 mg/mL for Co-Q_9;_ and CV intra-day 3.7%, CV inter-day 9.5%, LOD 0.012 mg/mL for Co-Q_10_). Plasma levels of retinol, tocopherols, ß-carotene, Co-Q_9_ and CoQ_10_ were assayed by high pressure liquid chromatography coupled to mass spectrometry (HPLC-MS), using methanol 0.1% and isocratic formic acid as solvent, with a flow of 0.5 ml/min in a ACQUITY UPLCr BEH C18 50 mm column (internal diameter 2.1 mm and particle size 1.7μm). β-carotene was also determined after extraction with 1-propanol in a HPLC system attached to a multiwavelength ultraviolet detector set at 450 nm. All these compounds were identified by predetermining the retention times of individual standards [28].

Hemoglobin concentration was determined by use of Drabkin´s reagent [29] (Sigma D5941) (CV intra-day 2.4%; CV inter-day 3.1%). The final concentration of Hb was adjusted to 5 mg/mL for antioxidant enzymes analyses. Catalase (CAT) activity was determined using de Aebi [30] (CV intra-day 15.5%, CV inter-day 17.4%) and it is is expressed as nmol/ (L · g Hb). SOD activity was assayed according to the methods of McCord & Fridovich (McCord & Fridovich 1969 [31]), using xanthine and xanthine oxidase to generate superoxide radicals. These radicals oxidize the cytochrome c, generating color measured at 450 nm. The presence of SOD competes with cytochrome c and blocks color generation m**e**asured at 450 nm (CV intra-day 5.1%, CV inter-day 7.0%). Data are expressed as U/mg Hb. GR was determined by measuring the rate of reduced nicotinamide adenine dinucleotide phosphate (NADPH) oxidation in the presence of oxidized glutathione (GSSG) according to Carlberg and Mannervik [32] (CV intra-day 8.0%, CV inter-day 10.3%). The results are expressed as U/g Hb. Finally, GPX was analyzed at 340 mn using the procedure developed by Flohé and Günzler [33] with tert-butyl hydroperoxide as substrate (CV intra-day 7.8%, CV inter-day 12.9%). The results are expressed as U/g Hb.

Urinary biomarkers 15-isoprostane F2t (F2-Iso) and 8-Hydroxydeoxyguanosine (8-OHdG) were determined using ELISA assay kits (Oxford Biomedical Research, Inc., Catalog # EA84.102606, Michigan, USA and JaICA, Nikken SEIL Co., Ltd, Catalog# IM-KOGHS 040914E, Shizuoka, Japan, respectively).

### Data Handling and Statistical Analyses

Data were organized and recorded in an SPSS version 20.0 database in order to run all the statistical analyses. Normality of variables was assessed using the Kolmogrov-Smirnov test. Descriptive statistics are presented as median, 95% CI and range. We ran the Spearman correlation coefficients according to the distribution of the sample. When the Spearman test gave a significant correlation coefficient (p <0.05) or one with a tendency to be significant (p >0.05 to <0.10), we ran goodness-of-fit models of SPSS in order to detect any improvement in the relation. Multiple regression backward stepwise analyses were performed to develop models to predict values of the urinary oxidative biomarkers F2-Iso and 8-OHdG from the antioxidant defense system parameters measured in the present study (SOD, CAT, GPX and GSR, and tocopherols, retinol, β-carotene and coenzymes Q_9_ and Q_10_) using the same software. Durbin-Watson statistics were used to assess whether the assumption of independent errors for the variables was tenable. ANOVA testing was done to determine whether the selected model was significantly better at predicting the outcome than using the mean as a “best guess”. The F represents the ratio of the improvement in the prediction as a result of fitting the model relative to the inaccuracy that still exists in the model. Variance inflation factor (VIF) and tolerance statistics were obtained to assess whether there was some co-linearity among the independent variables [34].

## Results

### Characteristics of the Participants

**Fig 1:**
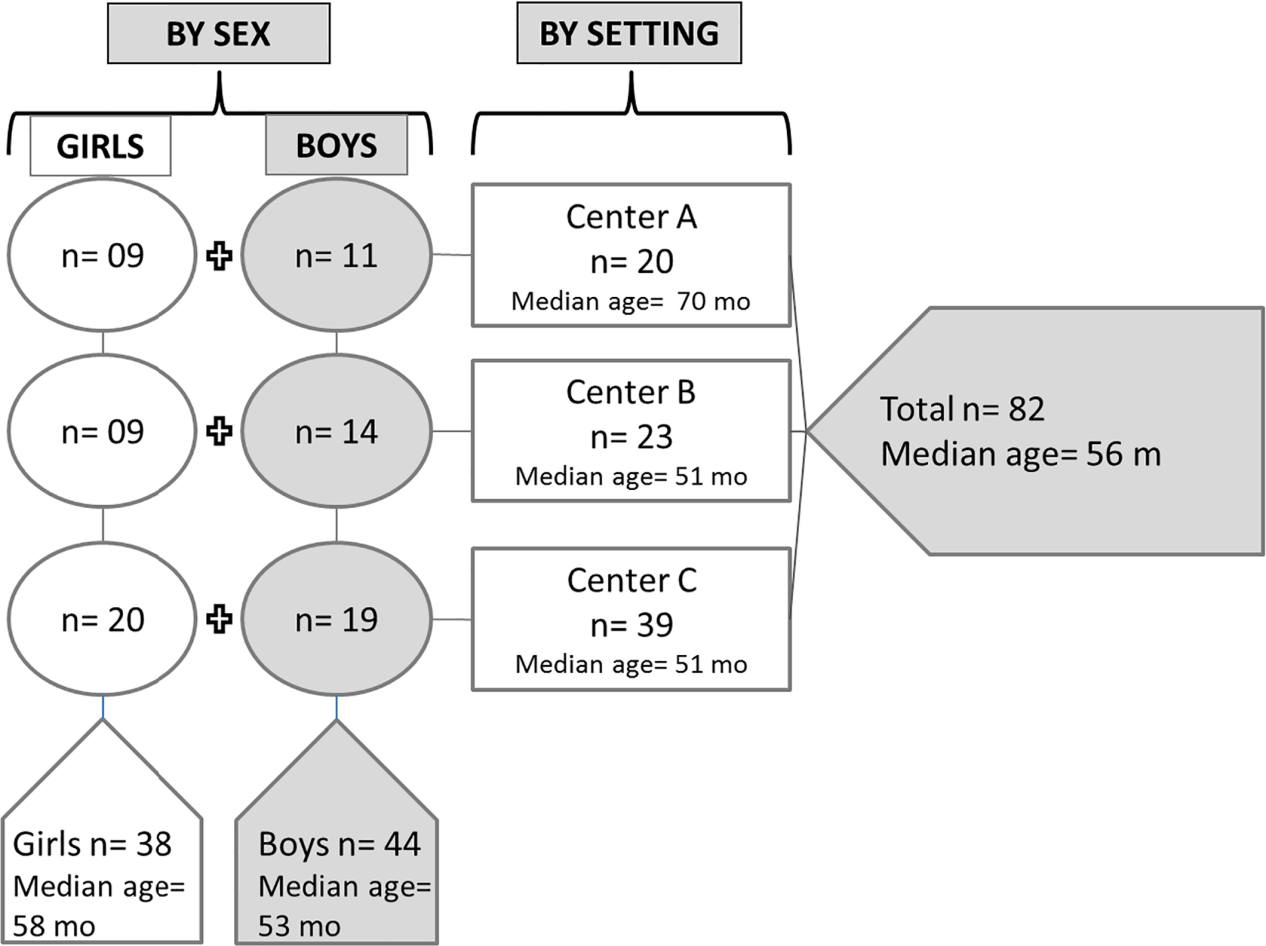
Characteristics of the Subjects, by setting and sex.

Of the 87 children enrolled in the study as a whole, binary samples were available variously in from 78 to 82 cases. In this binary sub-sample, 38 participants were girls, and 44 were boys. The ages ranged from 23 to 81 mo, with a mean of 55 ± 16 mo, and a median of 56 mo. **Fig 1** illustrates the sex- and age-distribution of the children for each of the three day-care centers, and provides data on the median ages by sex.

### Descriptive Statistics of the Biomarkers Concentration

**Table 1** shows the median, 95% confidence interval, and minimum and maximum values for each of the 11 biomarkers, as well as normalized values for the urinary oxidative stress biomarkers, adjusted for urinary creatinine.

**Table 1:** Descriptive Statistics of the Biomarkers Concentration.

Biomarker	N	Median	95% CI	Min-Max	Reference criteria
*Urinary oxidative stress biomarkers*					
F2-Iso (ng/mL)	78	1.75	1.93–2.81	0.50–11.80	—
8-OHdG (ng/mL)	78	5.69	6.26–9.80	0.55–38.61	—
F2-Iso (ng/mg creatinine)	78	5.86	6.15–9.47	2.21–52.03	>3.0
8-OHdG (ng/mg creatinine)	78	18.24	18.62–26.03	3.52–86.30	>8.4
*Antioxidant enzymes*					
CAT Activity (nmol/seg/g Hb)	82	6.68	6.46–7.05	4.31–9.91	—
SOD Activity (U/g Hb)	82	1.57	1.41–1.69	0.24–2.78	—
GSR Activity (μmol/min/g Hb)	82	2.82	2.67–2.93	1.79–4.06	—
GPX Activity (μU/g Hb)	82	6.05	5.57–6.41	0.00–11.99	—
*Antioxidant nutrients*					
Retinol (μg/dL)	81	21.8	20.8–23.5	11.1–44.1	30–95
Tocopherols (mg/L)	81	3.94	3.81–4.26	2.05–6.66	5–20
β-carotene (mg/L)	81	0.60	0.67–0.94	0.11–2.77	—
Co-Q_9_ (mg/L)	81	0.04	0.04–0.05	0.04–0.06	0.007–0.037
Co-Q_10_ (mg/L)	81	0.20	0.20–0.22	0.15–0.32	0.041–1.55

F2-Iso = 15-isoprostane F2_t_; 8-OHdG = 8-hydroxy-deoxy-guanosine; CAT = catalase; SOD = superoxide dismutase; GSR = glutathione reductase; GPX = glutathione peroxidase

### Geographic Selectivity of the Biomarker concentration

Shown in **Table 2** are the median values and 95% CI for the 13 biomarker expressions used in the study compared across the three geographical sites of the study. As compared by a three-way Kruskal-Wallis test, there were significant differences by site for all biomarkers, with the superscript “a” and the upward arrow indicating the highest in the row, and the superscript “b” and the downward arrow indicating the lowest. By a priori logic, the higher levels of urinary oxidative stress biomarkers and the lower concentrations of anti-oxidant vitamins were considered unfavorable, and presented in bold font. There is no a priori assignment of anti-oxidant enzyme activities. As seen by the accumulation of bold font, the marginal-urban center “B” was unfavorable for all nine relevant biomarkers. By contrast, the semi-urban center “A” had the favorable direction in eight of the nine indicators.

**Table 2:** Comparisons of oxidation and anti-oxidation biomarkers concentrations and activities by setting.

Biomarker	Center A		Center B		Center C		
	(semi-urban [n = 18])		(marginal-urban [n = 23])		(rural [n = 32]) Median [95% CI]		p-value*
	Median [95% CI]		Median [95% CI]				
8-OHdG (ng/mL)	4.33^a^ [3.61–5.60]	↓	**7.99**^**b**^ [6.96–14.03]	↑	5.10^ab^ [4.84–11.41]	—	0.044
8-OHdG (ng/mg creatinine)	13.90^a^ [11.95–15.87]	↓	**22.2**^**b**^ 19.98–38.98	↑	**18.88**^**b**^ [17.01–27.95]	↑	0.004
F2-Iso (ng/mL)	0.88^a^ [0.80–1.14]	↓	**3.29**^**b**^ [2.68–4.87]	↑	1.72^c^ [1.58–2.55]	—	<0.001
F2-Iso (ng/mg creatinine)	2.72^a^ [2.63–3.43]	↓	**8.47**^**b**^ [7.71–16.48]	↑	5.85^c^ [5.28–8.96]	—	<0.001
CAT (nmol/seg/ g Hb)	5.97^a^ [5.38–6.37]		7.68^b^ [7.20–8.35]		6.75^a^ [6.19–6.99]		<0.001
SOD (U/g Hb)	0.60^a^ [0.52–0.76]		1.57^b^ [1.46–1.82]		1.95^b^ [1.77–2.10]		<0.001
GSR (umol/min/g Hb)	2.21^a^ [2.18–2.73]		3.37^b^ [3.08–3.48]		2.74^a^ [2.50–2.87]		<0.001
GPX (μU/g Hb)	5.68^ab^ [5.33–6.37]		4.91^a^ [4.37–6.02]		6.57^b^ [5.83–7.38]		0.020
Retinol (mg/dL)	23.34^a^ [21.42–24.68]	↑	**16.26**^**b**^ [15.31–19.39]	↓	23.98^a^ [21.53–24.85]	↑	<0.001
Tocopherols (mg/L)	4.30^a^ [4.06–4.78]	↑	**3.35**^**b**^ [2.94–3.53]	↓	4.21^a^ [3.89–4.58]	↑	<0.001
β-carotene (mg/L)	1.65^a^ [1.38–1.92]	↑	**0.58**^**b**^ [0.51–0.75]	↓	**0.39**^**b**^ [0.36–0.65]	↓	<0.001
CO-Q_9_ (mg/L)	0.044^a^ [0.043–0.047]	↑	**0.043**^**b**^ [0.042–0.044]	↓	0.043^ab^ [0.043–0.046]	—	0.020
CO-Q_10_ (mg/L)	0.20^ab^ [0.19–0.22]	—	**0.19**^**b**^ [0.18–0.20]	↓	0.21^a^ [0.21–0.23]	↑	0.004

*Across the rows, values not sharing the same superscript letter are statistically different by the Kruskal-Wallis test.

The upward arrow represents the highest value(s) in the cross-row set, whereas the downward arrow represents the lowest value(s). The hyphen represents the intermediary value in a set. Such analysis was not made for antioxidant enzymes, as they do not have a reference value.

The bold font indicates the least favorable median value. Anti-oxidant enzymes have no a priori assignment for favorability.

### Hemimatrix of Linear Spearman Correlations for Inter-Biomarker Associations

**Fig 2:**
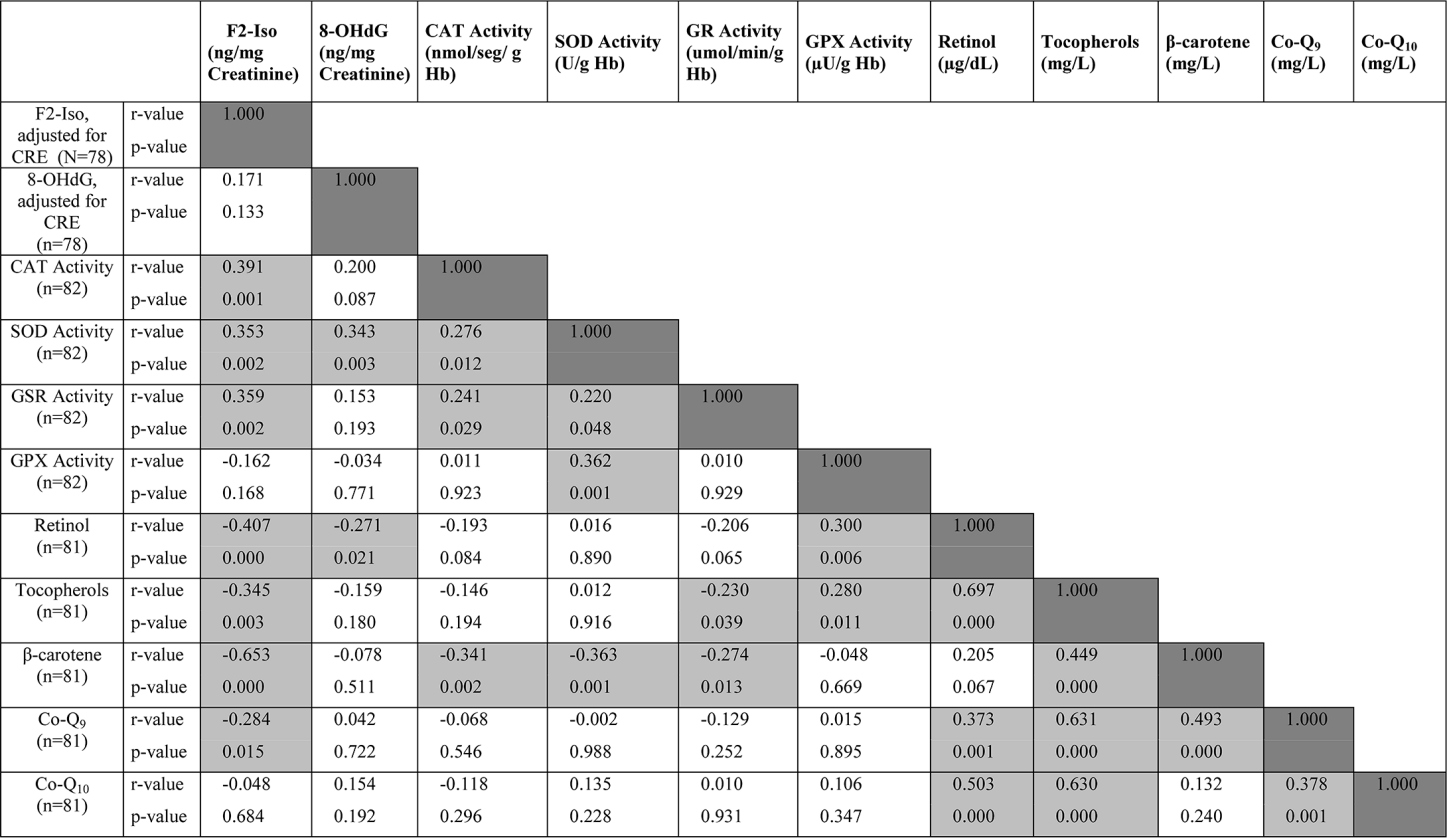
Spearman Correlation Coefficient Hemi-Matrix for Inter-Relationships of Biomarkers. The Spearman rank-order correlation coefficients Hemi-Matrix for mutual, cross inter-relationships of the 11 measured biomarkers are illustrated within the 66 cells of pertinent reference. The dark-shaded cells represent the 11 auto-correlations. The remaining 55 cells illustrate the probability level for the corresponding Spearman r value. The 28 medium-shaded cells have statistically-significant associations, whereas the 27 clear cells have non-significant associations. Corresponding units of concentration or activity are given with the biomarkers along the horizontal axis; n values are given in the vertical axis.

**Fig 2** shows the Spearman correlation coefficients for 55 binary inter-biomarker associations among the 11 variables including 2 urinary oxidative stress biomarkers, 4 rbc antioxidant enzymes and 5 plasma antioxidant compounds. Of these, 28 (51%) met the 5% statistical significance criterion. The strongest significance was found for the association between retinol and tocopherols (r = 0.697, p<0.001) and the weakest between SOD and GSR (r = 0.220, p = 0.048).

### Comparison of Goodness-of-Fit Correlations for Inter-Biomarker Associations

**Table 3** lists the 28 significant binary associations as analyzed by Spearman rank-order correlation and the corresponding goodness-of-fit correlation coefficient with the appropriate curve form. In 19 cases of the series (70%), the r value was improved or remained the same, whereas in 8 cases (30%) the goodness-of-fit r-value was lower than provided by Spearman analysis. The associations showing the greatest improvements for the strength of correlation were 8-OHdG adjusted for Creatinine and retinol (+75%) and SOD and β-carotene (+75%).

**Table 3:** Comparison of Spearman and Non-Linear Correlation Coefficients in Inter-Biomarker Significant Associations.

X-Axis	Y-Axis	Spearman r-value	Goodness-of-fit r-value	Best model curve form
F2-Iso adjusted for Creatinine	CAT	0.391	0.363	Power
	SOD	0.353	0.410	Sigmoid
	GSR	0.359	0.340	Exponential
	Retinol	-0.407	0.621	Cubic
	Tocopherols	-0.345	0.511	Cubic
	β-carotene	-0.653	0.621	Power
	Co-Q_9_	-0.284	0.326	Quadratic
8-OHdG adjusted for Creatinine	SOD	0.343	0.385	Cubic
	Retinol	-0.271	0.474	Cubic
CAT	SOD	0.276	0.282	Linear
	GSR	0.241	0.242	Exponential
	β-carotene	-0.341	0.347	Linear
SOD	GSR	0.220	0.235	Exponential
	GPX	0.362	0.295	Cubic
	β-carotene	-0.363	0.634	Cubic
GSR	Tocopherols	-0.230	0.209	Power
	β-carotene	-0.274	0.271	Power
GPX	Retinol	0.300	0.387	Cubic
	Tocopherols	0.280	0.344	Cubic
Retinol	Tocopherols	0.697	0.719	Cubic
	Co-Q_9_	0.373	0.453	Cubic
	Co-Q_10_	0.503	0.539	Cubic
Tocopherols	β-carotene	0.449	0.485	Sigmoid
	Co-Q_9_	0.631	0.679	Cubic
	Co-Q_10_	0.630	0.702	Cubic
β-carotene	Co-Q_9_	0.493	0.478	Cubic
Co-Q_9_	Co-Q_10_	0.378	0.367	Sigmoid

The r-value was improved or remained the same in 19 cases of the series, whereas the goodness-of-fit r-value was lower than Spearman’s in 8 cases.

There were 5 correlation coefficients in the probability range of 0.051 to 0.100 including those between 8-OHdG and SOD, CAT and retinol, GSR and retinol, β-carotene and retinol, and 8-OHdG and GSR. Only the latter two associations in this series achieved significance at p<0.05 when applying the goodness-of-fit model.

### Multiple Regression Models

**Table 4** shows the coefficients of the multiple regression models for the dependent variables urinary F2-isoprostanes and 8-hydroxy-deoxy-guanosine adjusted for creatinine using as independent variables the antioxidant defense system parameters measured in the present study (red blood cells activities of SOD, CAT, GPX and GSR, and plasma concentrations of tocopherols, retinol, β-carotene and coenzyme Q_9_ and Q_10_). Plasma retinol and β-carotene were independent predictors of F2-Iso, accounting for about 25% of the total variability, whereas plasma retinol and Co-Q_10_ were independent predictors of 8-OHdG accounting for 22% of the variability.

**Table 4:** Coefficients of the multiple regression model for the dependent variables of urinary F2-Iso and 8-OHdG, adjusted for creatinine, using as independent variables the antioxidant defense system parameters measured in the present study (SOD, CAT GPX and GSR, and plasma concentrations of tocopherols, retinol, β-carotene and Co-Q_9_ and Co-Q_10_).

		Unstandardized Coefficients		Standardized Coefficients			95.0% Confidence Interval for B	
No.	Model	B	Std. Error	Beta	t	Sig.	Lower Bound	Upper Bound
*Dependent variable*: F2-Iso adjusted for creatinine*								
8	(Constant)	20.229	3.286		6.156	<0.001	13.676	26.783
	Retinol (mg/dL)	-0.431	0.157	-0.296	-2.742	0.008	-0.744	-0.117
	β-carotene (mg/L)	-4.000	1.311	-0.330	-3.050	0.003	-6.616	-1.385
*Dependent Variable*: 8-OHdG adjusted for creatinine**								
7	(Constant)	5.471	12.439		0.440	0.661	-19.344	30.287
	SOD activity (U/g Hb)	5.823	2.748	0.227	2.119	0.038	0.341	11.306
	Retinol (mg/dL)	-1.290	0.393	-0.389	-3.284	0.002	-2.074	-0.506
	Co-Q_10_ (mg/L)	173.094	61.196	0.335	2.829	0.006	51.012	295.176

* r^2^ = 0.255; r = 0.505 (n = 73)

** r^2^ = 0.219; r = 0.468 (n = 73)

**Fig 3:**
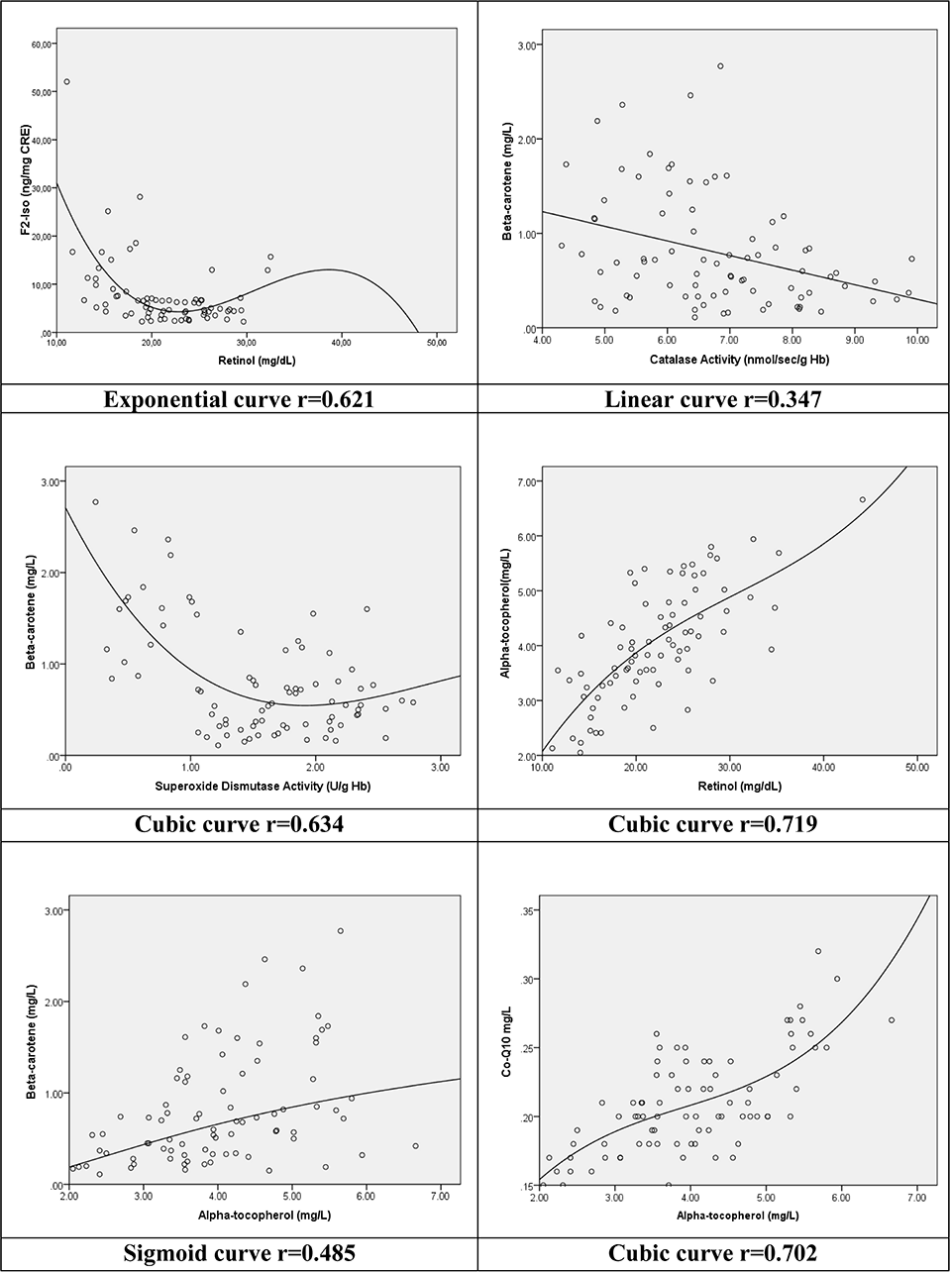
Selected associations between variables with the superimposition of the Goodness-of-Fit curve form and the correlation coefficient “r” for the model’s equation.

**Fig 3** illustrates 6 selected goodness-of-fit association curves showing the different curve-forms that appeared with the transformed regression model. These include three with cubic form and one each with exponential, sigmoid and linear forms as the result of the goodness-of-fit adaptation. Overall, the cubic configuration dominated with 14 (52%) of all transformations performed.

## Discussion

The underlying research hypothesis of this field survey was that the status of oxidative stress and other diagnostic domains would show substantial heterogeneity across the sample, despite the narrow age range and a common institutional menu. The findings amply confirm the notion of wide, inter-individual variance. At the second level, however, we observe broad, multiple and harmonious associations among antioxidant micronutrients, antioxidant enzymes and resulting free radical damage to lipid and nuclear cells constituents. Although one can never impute causality nor confirm direction of causality merely from associations, this opens up the suggestion that the control of low-grade oxidative stress, indeed, behaves like an interactive system within the organism. It has been suggested [35] that a number of adverse environmental exposures or chronic nutrient deficiencies or illnesses, common to the unsanitary and impoverished conditions of low-income settings, can induce or exacerbate cellular oxidation. Of special interest is the existence of indoor smoke contamination of the Western Highlands homes, which can provoke oxidative stress [36]. It was in fact, the scheme of common generalized environmental stressors that framed the rationale for our previous publication [25] examining the interaction of inflammatory biomarkers in these same subjects (**S1 Article**).

Given that the subjects came from residential settings as described above, with high levels of poverty and low levels of hygiene, it would not be surprising to find values of the biomarkers mildly outside of the normative ranges. For the urinary biomarkers of oxidative stress there are no established normal values for children. Using the normative range for F2-Iso, adjusted for creatinine, given as 3.0 ng/mg of creatinine by the manufacturers in the kit (Source: Oxford Medical Research manual), 66 subjects (85%) are above the upper value, when similarly adjusted. Also, all our subjects had higher values than the mean of 0.7 ng/mg of creatinine based on 39 healthy adolescent controls as published earlier, when compared in common units [37]. When comparing our results to normative values 8.4 ng/mg of creatinine for 8-OHdG provided with the used kit (Source: JaICA manual), 70 of our subjects (90%) had values above the adult mean value adjusted for creatinine concentration. Moreover, when compared to the median value of 9.5 ng/mg of creatinine for 14 healthy children aged 2 to 15 y of age [38], 68 of our subjects (87%) were above their median creatinine-adjusted value. Thus, it seems that a mild elevation in both urinary markers of oxidation is the rule in our population.

Unlike the urinary and plasma biomarkers in this study, for which normative ranges can be offered, activity expressions for anti-oxidant enzymes in red cell membrane cannot be treated or interpreted in a parallel manner. The large variation in assays and their expression across the literature precludes assigning diagnostic reference standard criteria, and they can best be interpreted in relative terms, as we have done in the present analysis of findings in **Tables 1** and **2.**

We measured circulating concentrations of five antioxidant nutrients. With respect to the reference system [39], the normal range of plasma circulating retinol is 30 to 95 μg/dL; in this context 75 of 81 samples (92%) would be considered low. This finding is surprising, as the most recent national nutrition survey [40] found the 6 to 59 mo population of Guatemala to be free of hypovitaminosis A; we suspect that there might be a calibration error in our analyses, but one that would not invalidate the relative rank-order among subjects in any way. The same reference source [39], provides the normative range from 5 to 20 mg/L for circulating levels of tocopherols; in our sample 64 of 81 samples (79%) are considered below the normal range. Variation in higher or lower levels of circulating lipids, which are the carriers of tocopherols, can distort the interpretation of vitamin E status [41,42]. Unfortunately, we do not have measurement of plasma cholesterol or triglycerides for this population. Nonetheless, at best, only about half of the low levels would normalize by adjustment [41], and even 40% prevalence of low tocopherols is an ominous finding.

The circulating levels of carotenoids are so dependent on the consumption of foods of plant origin for which no international references can or should be made. A previous longitudinal study of urban schoolchildren in Guatemala, conducted between February and May, documented a 3-fold increase in total carotene concentration as compared to other seasons, attributed to the mango season [43]. In the present study, blood was sampled from August through October, i.e. during the downslope of the annual ß-carotene cycle from native mangos.

According to Molyneux et al. [44], the normal range for Co-Q_10_ is 0.41 to 1.55 mg/L. With our values ranging from 0.15 to 0.32 mg/L, all subjects in this study would be considered to have low Co-Q_10_ status. However, with the reference range for Co-Q_9_ from 0.007 to 0.037 mg/L [44] and with our subjects’ distribution 0.040 to 0.060 mg/L, our lowest value is *above* the normative reference range.

After the description of biomarker values in the context of status assessment in **Table 1**, we examined the status across geographic sites. In terms of the hypothesis of mutual associations, the clustering of favorable and adverse diagnostic implications of the biomarkers, as shown in **Table 2**, adds another dimension to the confirmation. In this respect, the association is geographic, and those with the greatest evidence of oxidative stress as a group also have the lowest concentrations of the vitamins and vitamin-like constituents in their circulation.

Multiple comparisons to evaluate interactions always run the risk of spuriously significant correlations. In our case, with a 5% probability criterion, we would have expected to find 2 and 3 correlations with a p value of <0.05 by chance alone. With our finding of a total of 28, at least 25 interactions are likely to represent truly significant associations. In addition, the fact that the goodness-of-fit models are also significant and improved in their r value in 86% of the instances is additional evidence for the validity of the Spearman associations encountered to be truly significant, and not spurious.

Although causality cannot be deduced from association alone, assigning directionality in the regression model (Table 3) is illustrative. Of all of the biomarkers, adjusted F2-Iso had the widest range of cross associations, with 7 of the 10 companion biomarkers; only adjusted 8-OHdG, GPX and Co-Q_10_ did not associate with F2-Iso adjusted for creatinine. Plasma retinol and β-carotene were identified as independent predictors of adjusted F2-Iso after running the backwards multiple regression model. Tocopherols also showed 7 significant cross-associations. The multiple-regression model was also applied for adjusted 8-OHdG; and SOD activity, plasma retinol and Co-Q_10_ were the resultant independent predictors.

Clearly, excessive oxidation is suggested to be associated with an increase in the urinary biomarkers of oxidative stress F2-Iso and 8-OHdG. Interpretation of the other biomarkers is not so straightforward. In theory, the response to oxidative stress could deplete a vitamin or affect the activities of enzyme biomarkers or produce a compensatory and protective increase. In the present experience, urinary excretion of oxidative biomarkers associates directly with the activity of antioxidant enzymes and inversely with the vitamin concentration. This association suggests that the enzymatic response is compensatory, whereas the antioxidant nutrients may be being consumed by the oxidation process. This interpretation is further confirmed by the fact the relationships than exist between ADS enzymes and non-enzymatic antioxidants are universally inverse.

### Strengths and limitations of the study

We acknowledge certain strengths and weaknesses of design and execution of the study. The principal strength is that it deals with childhood, with children being of preschool age. In theory, the settings with similar dietary offering should control one important variable, and narrow overall variance. We were ambitious enough to attempt 24-h urine collection in the design for young children, with relative success in obtaining complete collections. We recognize, however, that we did not obtain full adherence in this effort due to the young age of the subjects; thus reliance on concentration-related variables for urinary analytes had to supersede that on total one-day output. An additional strength is the possibility to relate a large variety of oxidative and antioxidant biomarkers of different classes and collected from different body fluids to each other, although, we have not considered or measured a variety of other endogenous and exogenous small-molecule antioxidants such as oxidized and reduced glutathione, and alpha-lipoic acid, ferritin, uric acid, bilirubin, metallothioneine, L-carnitine and melatonin [45]. In terms of limitations, however, our central thesis of interactions is based on geographical clustering and rank-order correlations and we remain aware that a conclusion for causality can never be derived from associations alone. The use of an ELISA method for urinary 8-OHdG provides a reasonable approach for comparisons made with the same kit, but we realize that the external validity is inferior to methods based on chromatography [46]. Finally, there are some inconsistences between the retinol status observed here and other experiences seen in Guatemala, and the lack of adjustment of tocopherols levels for circulating lipids may be producing an overestimation of deficiency for this nutrient.

### Conclusion

In conclusion, the largely common menu offering at the daycare centers during 5 days per week was no guarantee of the nutritional or a physiological uniformity across a sample of low-income preschoolers. Nutritional biomarkers varied widely, with many falling below the cutoff for adequate status. The best available explanation for mildly elevated urinary biomarkers of oxidative stress would be exposure to the constellation of household and community exposures originating in the poor sanitation, hygiene, and air and water quality of the areas of study. Most strikingly, however, are apparent patterns of harmonious interactions among the elements of the oxidation-antioxidation systems within a setting that presents a variety of mild to moderate oxidative stress from the conditions of daily existence.

## Supporting Information

S1 STROBE Checklist STROBE statement for observational studies. (DOC)

S1 Article Article by Soto-Méndez, et al. Associations among Inflammatory Biomarkers in the Circulating, Plasmatic, Salivary and Intraluminal Anatomical Compartments in Apparently Healthy Preschool Children from the Western Highlands of Guatemala. PLoS ONE DOI:10.1371/journal.pone.0129158 June 15, 2015.
(PDF)
