# Correction: Strong Associations Exist among Oxidative Stress and Antioxidant Biomarkers in the Circulating, Cellular and Urinary Anatomical Compartments in Guatemalan Children from the Western Highlands

**DOI:** 10.1371/journal.pone.0149740

**Published:** 2016-02-12

**Authors:** María J. Soto-Méndez, Concepción M. Aguilera, María D. Mesa, Laura Campaña-Martín, Victoria Martín-Laguna, Noel W. Solomons, Klaus Schümann, Ángel Gil

In the caption for Table 2, a portion of footnote text is incorrectly included in the manuscript text. Please see the corrected Table 2 here.

**Table 2:** Comparisons of oxidation and anti-oxidation biomarkers concentrations and activities by setting.

Biomarker	Center A		Center B		Center C		
	(semi-urban [n = 18]) Median [95% CI]		(marginal-urban [n = 23]) Median [95% CI]		(rural [n = 32]) Median [95% CI]		p-value*
8-OHdG (ng/mL)	4.33^a^ [3.61–5.60]	↓	**7.99**^**b**^ [6.96–14.03]	↑	5.10^ab^ [4.84–11.41]	-	0.044
8-OHdG (ng/mg creatinine)	13.90^a^ [11.95–15.87]	↓	**22.2**^**b**^ 19.98–38.98	↑	**18.88**^**b**^ [17.01–27.95]	↑	0.004
F2-Iso (ng/mL)	0.88^a^ [0.80–1.14]	↓	**3.29**^**b**^ [2.68–4.87]	↑	1.72^c^ [1.58–2.55]	-	<0.001
F2-Iso (ng/mg creatinine)	2.72^a^ [2.63–3.43]	↓	**8.47**^**b**^ [7.71–16.48]	↑	5.85^c^ [5.28–8.96]	-	<0.001
CAT (nmol/seg/ g Hb)	5.97^a^ [5.38–6.37]		7.68^b^ [7.20–8.35]		6.75^a^ [6.19–6.99]		<0.001
SOD (U/g Hb)	0.60^a^ [0.52–0.76]		1.57^b^ [1.46–1.82]		1.95^b^ [1.77–2.10]		<0.001
GSR (umol/min/g Hb)	2.21^a^ [2.18–2.73]		3.37^b^ [3.08–3.48]		2.74^a^ [2.50–2.87]		<0.001
GPX (μU/g Hb)	5.68^ab^ [5.33–6.37]		4.91^a^ [4.37–6.02]		6.57^b^ [5.83–7.38]		0.020
Retinol (mg/dL)	23.34^a^ [21.42–24.68]	↑	**16.26**^**b**^ [15.31–19.39]	↓	23.98^a^ [21.53–24.85]	↑	<0.001
Tocopherols (mg/L)	4.30^a^ [4.06–4.78]	↑	**3.35**^**b**^ [2.94–3.53]	↓	4.21^a^ [3.89–4.58]	↑	<0.001
β-carotene (mg/L)	1.65^a^ [1.38–1.92]	↑	**0.58**^**b**^ [0.51–0.75]	↓	**0.39**^**b**^ [0.36–0.65]	↓	<0.001
CO-Q_9_ (mg/L)	0.044^a^ [0.043–0.047]	↑	**0.043**^**b**^ [0.042–0.044]	↓	0.043^ab^ [0.043–0.046]	-	0.020
CO-Q_10_ (mg/L)	0.20^ab^ [0.19–0.22]	-	**0.19**^**b**^ [0.18–0.20]	↓	0.21^a^ [0.21–0.23]	↑	0.004

*Across the rows, values not sharing the same superscript letter are statistically different by the Kruskal-Wallis test. The upward arrow represents the highest value(s) in the cross-row set, whereas the downward arrow represents the lowest value(s).

The hyphen represents the intermediary value in a set. Such analysis was not made for antioxidant enzymes, as they do not have a reference value. The bold font indicates the least favorable median value. Anti-oxidant enzymes have no a priori assignment for favorability.
